# Functions of membrane proteins in regulating fruit ripening and stress responses of horticultural crops

**DOI:** 10.1186/s43897-024-00111-5

**Published:** 2024-09-24

**Authors:** Daoguo Chen, Yuhan Liu, Yong Chen, Boqiang Li, Tong Chen, Shiping Tian

**Affiliations:** 1 State Key Laboratory of Plant Diversity and Specialty Crops, Institute of Botany, Chinese Academy of Sciences, Nanxincun 20, Xiangshan, Haidian District, Beijing, 100093 China; 2 China National Botanical Garden, Beijing, 100093 China; 3 University of Chinese Academy of Sciences, Beijing, 100049 China

**Keywords:** Endomembrane proteins, Fruit, Ripening, Plasma membrane proteins, Resistance

## Abstract

Fruit ripening is accompanied by the development of fruit quality traits; however, this process also increases the fruit’s susceptibility to various environmental stresses, including pathogen attacks and other stress factors. Therefore, modulating the fruit ripening process and defense responses is crucial for maintaining fruit quality and extending shelf life. Membrane proteins play intricate roles in mediating signal transduction, ion transport, and many other important biological processes, thus attracting extensive research interest. This review mainly focuses on the functions of membrane proteins in regulating fruit ripening and defense responses against biotic and abiotic factors, addresses their potential as targets for improving fruit quality and resistance to environmental challenges, and further highlights some open questions to be addressed.

## Introduction

Fruit ripening is an important developmental stage that simultaneously occurs with the formation of fruit quality traits, i.e., color, texture, and aroma (Chen et al. 2020a). While resistance to biotic and abiotic stress is also a crucial trait affecting ultimate quality and commodity of fruit at pre-harvest and post-harvest stages (Zhang et al. 2021a). Accumulating evidence has been reported that these two processes are regulated by sophisticated mechanisms, in which membrane proteins have gradually received extensive research interest owing to their pivotal roles (Ji et al. 2020a, 2023).

According to the fluid-mosaic model, all biological membranes are composed of phospholipid bilayer and proteins, for which proteins make up about half of the entire mass. These membrane proteins are mainly categorized into integral, peripheral, and anchored proteins. Integral proteins come across the entire thickness of the phospholipid bilayer, and most receptors (receptor-like kinases, receptor-like proteins) are representatives in this type. In contrast, peripheral proteins are bound to the membrane by non-covalent bonds, while anchored proteins are bound to the membrane surface via covalently attached lipid molecules (Lazar et al. 2003). Alternatively, membrane proteins may also be simply categorized into plasma membrane proteins and endomembrane proteins according to their subcellular localization (Chen et al. 2018). This review will focus on the advances in identifying functions of membrane proteins in regulating fruit ripening and stress responses, which may provide further insights into the mechanisms underlying fruit quality formation and resistance, thereby guiding future efforts to prolong shelf-life and reduce postharvest loss.

## Plasma membrane proteins in fruit ripening and stress responses

As the frontier separating cellular structures from the external environment, the plasma membranes (PM) play irreplaceable roles in maintaining cell morphology, controlling substance entry and exit, signal transduction, etc. Many essential functions of the PMs are carried out by their proteinaceous complexes, including molecular transport, cell–cell interactions, ligand binding, signal transduction, and environmental sensing (Zhang et al. 2008). Recent studies have shown that PM-associated proteins are involved in regulating both fruit ripening and resistance.

### FERONIA (FER)

FER, one of the receptors-like kinases (RLKs) initially identified as a key player in the reception of pollen tubes during fertilization in *Arabidopsis* (Duan et al. 2020; Zhang et al. 2021b). Recently, this protein kinase is involved in various signaling pathways, particularly in the regulation of developmental processes and stress responses (Ji et al. 2020b).

**Fig. 1:**
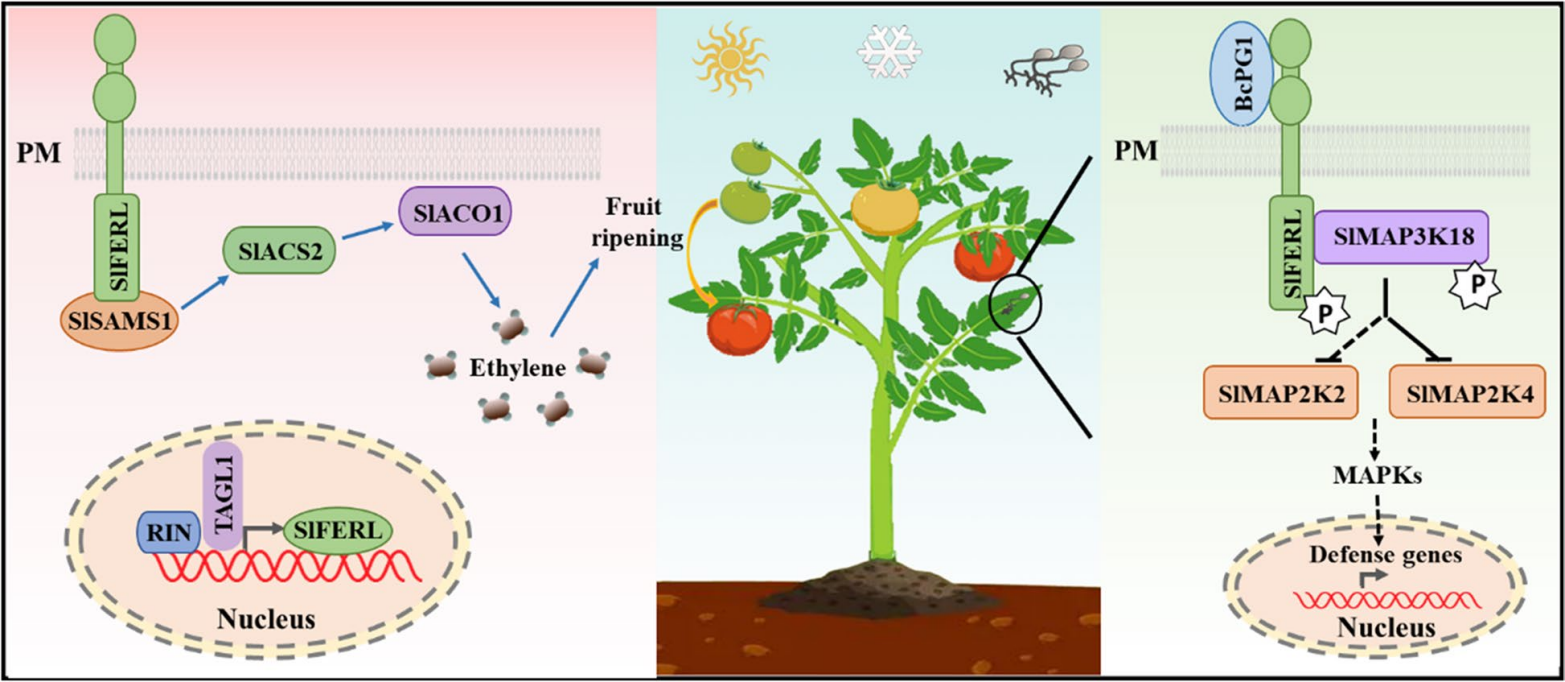
The working model for SlFERL in regulating fruit ripening and defense responses. Upon transcriptional activation by RIN-TAGL1, SlFERL recruited and interacted with SlSAMS1 on the PM, further regulated ethylene biosynthesis and fruit ripening. Meanwhile, SlFERL recognized BcPG1, a virulence protein secreted by *B. cinerea*, further phosphorylated SlMAP3K18 to initiate MAPK cascade. Both SlMAP2K2 and SlMAP2K4 substantially contribute to the defense responses of tomato to *B. cinerea*. P, phosphorylation

Fruit are specialized organs with crucial roles in seed-bearing and dispersal for angiosperms, while hormones are important factors affecting fruit ripening (Lazar et al. 2003). Several lines of evidence have shown that FER also regulates fruit ripening via modulating hormone signaling in both climacteric and non-climacteric fruit. In terms of climacteric fruit with almost concurrent ethylene (ET) and respiration peaks during ripening, ET serves as a core regulator (Chen et al. 2020a). FERONIA Like (FERL) physically interacts with *S*-adenosyl-Met synthetase (SAMS), a key enzyme in the ET biosynthesis pathway, to regulate ET production both in apple (*Malus* × *domestica*) and tomato (*Solanum lycopersicum*). Overexpression of *MdFERL6* in apple callus inhibited ET production, and heterologous expression of the *MdFERL6* in tomato fruit also suppressed ET production and delayed ripening (Jia et al. 2017b). In contrast, knockdown of *SlFERL* by RNA interference resulted in delayed tomato fruit ripening, while overexpression of *SlFERL* significantly accelerated the ripening process (Ji et al. 2020a). These different results may be attributed to the different regulatory machinery of FERL in different fruits or existence of the homologs potentially redundant functions. Among the 17 members in *MdFERL* family in the genome of apple, *MdFERL6* was expressed at high level during early fruit development, but the expression dramatically declined when fruit ripening commenced (Jia et al. 2017b). On the contrary, the expression of *SlFERL* increased persistently during tomato fruit ripening (Ji et al. 2020a). Upon the onset of fruit ripening, the MADS-box transcription factor RIPENING INHIBITOR (RIN) and tomato AGAMOUS-LIKE1 (TAGL1) could bind to the promoter region of *SlFERL* to further activate its expression transcriptionally (Fig. 1) (Ji et al. 2020a). SlFERL further recruits *S*-adenosyl-L-methionine synthetase 1 (SlSAMS1) to plasma membrane and modulate its activity to produce *S*-adenosyl-methionine, which serves as an ET precursor and possibly methyl group donor for DNA methylation (Ji et al. 2020a). However, this study did not fully explain the mechanism underlying the interaction between SlFERL and SlSAMS1. It would be interesting to ascertain whether SlFERL modulates the enzymatic activity of SlSAMS1 by phosphorylation modification. In contrast, abscisic acid (ABA) acts as an important factor for the ripening of non-climacteric fruit. A FER-like receptor kinase in strawberry, *FaMRLK47*, physically interacts with a negative regulator of ABA signaling, *FaABI1*, to activate the expression of ripening-related genes. Overexpression of *FaMRLK47* delayed strawberry fruit ripening, but resulted in an increased sucrose content through promoting the expression of *FaSS* and *FaSPS* (Jia et al. 2017a). Firmness and sugar-acid ratio are important determinants of fruit taste and quality, and variations in firmness and sucrose content may contribute to the emergence of fruit with distinctive flavors, which may be an excellent trait favored by farmers.

In addition to its roles in regulating fruit ripening in various species, FER is also important in responding to pathogen invasions (Liao et al. 2017). As a PM-localized RLK, FER is renowned for its proficiency in recognizing microbe- and pathogen-associated molecular patterns, thereby initiating a series of subsequent immune responses (Couto and Zipfel 2016; Tang et al. 2017). *Fusarium oxysporum*, a soil-borne fungal pathogen, can secrete Rapid Alkalinization Factor (RALF)1-like peptides that can mimic the function of plant-derived RALFs to interact with FER and further hijack the downstream signaling in its host plants, ultimately taking over host immunity response (Masachis et al. 2016; Thynne et al. 2017). Phenotypic analysis indicated that F-RALF caused growth arrest of roots, whereas the *fer-4* knockout mutant displayed enhanced resistance against *Fusarium* (Masachis et al. 2016). This phenomenon can be explained by the suppression of the channel activity of H^+^-ATPase 2 (AHA2) by FER, leading to subsequent extracellular alkalinization (Haruta et al. 2014). According to Haruta et al. (2014), this extracellular alkalinization facilitates *Fusarium* infection by stimulating the phosphorylation of Fmk1, a mitogen-activated protein kinase (MAPK) essential for the infection process. This phosphorylation event is a critical step in the signaling pathway that *Fusarium* uses to successfully establish infection in the host.

Interestingly, RALF-like peptides have also been reported in some phylogenetically distant groups of fungi. Similar cases have been documented for *F. graminearum* and *Meloidogyne incognita*, where FER negatively regulates plant immunity and cell growth (Zhang et al. 2020b). In these instances, the presence of RALF-like peptides from the pathogens can interact with FER, leading to suppression of the defense mechanisms of plants and promoting infection. Given the increased susceptibility of RNAi fruit to *B. cinerea*, it was recently demonstrated that the extracellular domain of SlFERL recognizes and interacts with the virulence protein BcPG1 (endopolygalacturonase1) secreted by *B. cinerea*, independent of the enzymatic activity of BcPG1 as a pectin-degrading enzyme (Ji et al. 2023). Interestingly, infiltration with purified recombinant BcPG1 did not induce cell death in wild-type tomato leaves but resulted in cell death in *slferl* mutant, indicating a potential role of SlFERL in regulating cell death. Alternatively, it could not be ruled out that the function of SlFERL in regulating cell death may be co-opted by *B. cinerea* by an unknown mechanism. In addition, SlFERL phosphorylates SlMAP3K18 and thus activates a MAPK cascade, modulating MAP2K4 protein level and activity and fine-tuning the defense responses of tomato to *B. cinerea* (Ji et al. 2023). It is noteworthy that other regulators functioning in FER signaling desensitization/attenuation are still not available up to now, which deserve further exploration.

FER receptor kinase at the crossroads of hormone signaling and stress responses, allowing plants to balance growth and defense (Liao et al. 2017; Zhang et al. 2023a). The mechanism of FER pleiotropic function still needs to be further elucidated.

### Remorins (REMs)

**Fig. 2:**
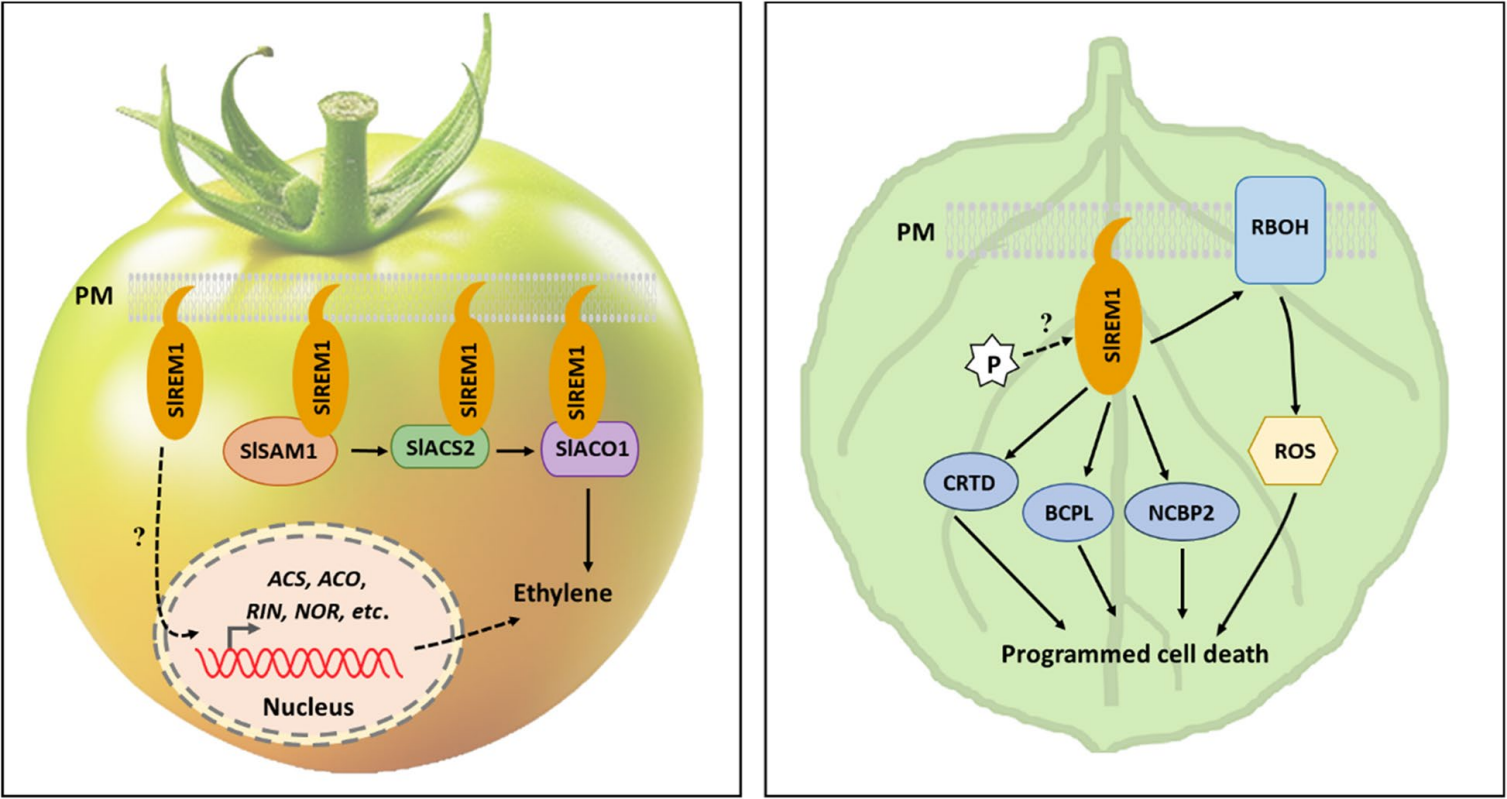
The working model for SlREM1 in regulating tomato fruit ripening and cell death. SlREM1 interacted with the key enzymes (namely SlSAM1, SlACS2 and SlACO1) involved in ethylene biosynthesis, to modulate ethylene production. SlREM1 also induced programmed cell death (PCD) by activating reaction oxygen species (ROS) production and up-regulating abundance of CYSTEINERICH AND TRANSMEMBRANE DOMAIN-CONTAINING PROTEIN A-LIKE (CRTD), BLUE COPPER PROTEIN-LIKE (BCPL), NUCLEAR CAPBINDING PROTEIN SUBUNIT 2 (NCBP2), and RBOHB

REMs are plant-specific PM-anchored proteins, which have been widely reported for their functions in various biological processes, including fruit ripening and stress resistance (Cai et al. 2020; Fu et al. 2018; Son et al. 2014). REMs have a highly conserved C terminus with a coiled-coil domain that is responsible for their PM localization and protein interactions, while the divergent N terminus harbors potential phosphorylation sites (Cai et al. 2020; Raffaele et al. 2007). The function of REMs on fruit ripening was first hinted by Raffaele et al. Indeed, the authors found that the overexpression of REM1 accelerated senescence in tomato seedlings in a degree, and the fruit in the REM over-accumulated line 2 (O2) also showed an accelerated ripening process unexpectedly (Raffaele et al. 2009). However, they did not provide a possible underlying mechanism to explain the phenotype. Until 2018, Cai et al. found that SlREM1 interacted with three key enzymes in the ET biosynthesis pathway, SAM1, ACS2 and ACO1, to positively regulate tomato fruit ripening (Fig. 2). Overexpression of *SlREM1* stimulated ET production and lycopene accumulation by up-regulating the key genes involved in ET production, lycopene biosynthesis and ripening regulation (Cai et al. 2018; Li et al. 2023). In addition, it was found that REM1 harbored a couple of potential ubiquitination sites and was degraded by the ubiquitin-26S proteasome system via an unknown mechanism, which may be responsible for the expression pattern of REM1 that increased at the breaker and orange stages, but decreased at the red ripe stage (Cai et al. 2018).

Besides the roles in regulating fruit ripening, REMs have been implicated in regulating ROS burst and cell death activity (Raffaele et al. 2009). Coincidentally, Cai reported that the overexpression of *SlREM1* increased the susceptibility of tomato leaves to *B. cinerea* and triggered cell death in *Nicotiana benthamiana* leaves (Cai et al. 2020). This overexpression also led to increased transcript levels of *N. benthamiana* RESPIRATORY BURST OXIDASE HOMOLOG (*NbRBOHB*) by a currently undetermined machinery, suggesting that SlREM1 may promote plant cell death through the activation of ROS production. Moreover, REM1 overexpression up-regulated the protein levels of some previously unidentified cell death regulators, such as cysteine-rich and transmembrane domain-containing protein A-like, blue copper protein-like, and nuclear cap-binding protein subunit 2, which may be resistance (R) proteins guarded by REM1. Interestingly, a recent study revealed that transient expression of *CaREM1.4* in pepper leaves triggered cell death and ROS accumulation (Zhang et al. 2023b), while knock-down of *CaREM1.4* in pepper down-regulated the expression of immunity-related genes and attenuated the resistance of pepper plants to *Ralstonia solanacearum*. As plants may perceive pathogens through receptor-like kinases in the PM or detect virulence proteins by resistance (R) proteins in the cytoplasm, thereby activating immune responses and triggering cell death (Yu 2020). These results not only add more experimental data to support the guard-guardee model for the function of REM in plant immunity, but also highlight the intricate interplay between PM proteins, ROS production, and plant immunity (Cai et al. 2020; Cesari 2018).

### Other plasma membrane proteins

Beside these two types of family proteins regulate both resistance and ripening, there are also some important proteins regulate fruit ripening and disease resistance.

### SWEETs

Sugars are primary metabolites providing building blocks and energy for cells, the sugar content determines fruit sweetness and ultimately fruit quality, while fleshy fruit are favored by consumers for the high sugar content (Chen et al. 2021; Jia et al. 2013; Kessenbrock et al. 2017). Sugar Will Eventually be Exported Transporters (SWEETs) are mainly localized at the PM (Anjali et al. 2020), whereby regulating sugar transport and accumulation together with sorbitol transporters (SOTs), sucrose transporters (SUTs) and monosaccharide transporters (MSTs) (Jeena et al. 2019). SlFgr, a member of the SWEET family in tomato, is a glucose transporter (Shammai et al. 2018). *SlFgr* overexpression reduced glucose levels and allowed for an increased fructose level, improving fruit flavor. During the development of *SlFgr* overexpression fruit, some ET-responsive transcription factors (such as Solyc05g052050 and Solyc12g056590), protein kinase genes involved in signal transduction (such as Solyc04g009900, Solyc05g056200 and Solyc04g074000), as well as some cell wall metabolism genes (such as Solyc02g093580 and Solyc03g097050), are differentially expressed, suggesting a likely effect on ripening-related metabolism (Shammai et al. 2018). In two varieties of the Ussurian pear (*Pyrus ussuriensis*), ‘Nanguo’ (NG) and its bud sport (BNG), BNG was sweeter than NG, which resulted from the higher expression of a sucrose transporter, *PuSWEET15* (Li et al. 2020b). PuWRKY31 positively regulated the expression of *PuSWEET15* by binding to its promoter. In BNG fruit, higher expression level of *PuWRKY31* was identified, which was associated with a higher level of histone acetylation in its promoter and coding sequence (CDS) regions. SWEETs were also involved in banana fruit development and ripening, as most *MaSWEETs* were expressed during banana ripening stages (Miao et al. 2017). During early stage of fruit development, the increase in *MaSWEET* expression augmented sugar transport ability, thereby enhancing quality and yield of banana fruit.

Beside the regulation on fruit quality, *SWEET* also act as susceptibility (*S*) genes in response to pathogen invasions. In plant-pathogen interactions, pathogens could secrete virulence proteins, described as transcription activator-like (TAL) effectors to hijack sugar transporters as a source of nutrition for themselves, leading to successful infection (Chen et al. 2021; Gupta 2020). These results have been well studied in *Arabidopsis* and rice. In *Arabidopsis thaliana*, the *SWEET* genes could be induced by *Pseudomonas syringae* pv. *tomato* DC3000, *Golovinomyces cichoracearum*, *Plasmodiophora brassicae* and *Pythium irregulare* (Chong et al. 2014; Ji et al. 2022). The *A. thaliana SWEET4* knockout mutant was more resistant to *Botrytis cinerea* (Chong et al. 2014). The *A. thaliana sweet11/sweet12* double mutants accumulated sugar in leaves, thereby triggering salicylic acid-mediate defense pathway to against the fungal pathogen *Colletotrichum higginsianum* (Gebauer et al. 2017). The rice *OsSWEET11-15* has been proven to provide nutrition, especially sugars, for *Xanthomonas oryzae* (Li et al. 2013; Zhou et al. 2015; Gao et al. 2018). After *X*. *oryzae* attack, specific TAL effectors were secreted from pathogens. These TAL effectors bind to specific effector-binding elements (EBEs) in the promoter region of the target *OsSWEET* genes. This interaction induces the up-regulated expression of the *OsSWEET* genes (Ji et al. 2022). In fruit, *B. cinerea* triggered a strong up-regulation of *VvSWEET4* expression in grapevine (*Vitis vinifera*), which could enhance hexose efflux and/or plant cell death, both being beneficial to fungal growth (Chong et al. 2014). In tomato, *SlSWEET15* also induced by *B. cinerea*, which may provide sugars to promote hyphal growth in the pre-necrotic stage of infection (Asai et al. 2016). Remarkably, some *SWEET* genes were down-regulated upon *B. cinerea* infection and others exhibited different expression patterns in different plant tissues, but their underlying functions still require further dissection (Asai et al. 2016; Breia et al. 2019).

### RBOHs

Plant respiratory burst oxidase homologs (RBOH) are closely related to ROS production, which is a key player in plant development and an important component for defense responses to pathogen infections (Sagi et al. 2004; Wang et al. 2019; Waszczak et al. 2018). RBOHs are involved in fruit development and ripening. *Lerboh1* antisense (M) plants showed abnormal petal number (more than six), fasciated styles and ovaries, and a high ratio of abnormal fruit (Sagi et al. 2004). During sweet pepper ripening, total CaRBOH activity significantly increased, which appears to be inhibited by NO-mediated post-translational modifications, especially S-nitrosation, Tyr-nitration and probably also by glutathionylation (Chu-Puga et al. 2019; González-Gordo et al. 2020). Besides its functions in ripening regulation, RBOHs are also essential for ROS accumulation in response to pathogen invasions (Li et al. 2015). SlRBOHB positively regulated the resistance to *B. cinerea*. Transient silencing of *SlRbohB* reduced the resistance to *B. cinerea*, while the *SlRbohB*-silenced plants showed higher ROS and lower expression of defense-related genes following *B. cinerea* infection. Another study reported that the silencing of *SlRBOH1* in tomato increased the root susceptibility to nematode and compromised BR-induced activation of MPK1/2 and MPK3 (Song et al. 2018). In a study employing chitin treatment, higher phosphorylation level of SlRBOHD was observed in the RIPK overexpression plants as compared to the knockout plants (Wang et al. 2022b). These results indicated that RIPK regulated ROS accumulation by phosphorylating SlRBOHD, thereby enhancing resistance of tomato to *B. cinerea* and *F. oxysporum*. Coincidently, CsRBOH2, a RBOH protein in *Citrus sinensis*, also plays a crucial role in regulating host resistance to citrus bacterial canker (CBC) by mediating ROS homeostasis (Li et al. 2024b). This regulation confers resistance to CBC in citrus plants. However, when *CsRBOH2* is knocked down, the resistance to CBC is significantly reduced, highlighting the importance of *CsRBOH2* in the defense mechanism against this disease (Li et al. 2024b).

## Proteins of endomembrane system contribute to fruit ripening and stress responses

Different from plasma membrane, endomembrane system refers to a series of membranous organelles interrelated structurally and functionally, such as endoplasmic reticulum (ER), Golgi apparatus, endosomes, secretory vesicles, etc. This intracellular membrane system greatly expands the surface area of the membrane and provides attachment sites for enzymes, which is conducive to metabolic reactions. Meanwhile, endomembrane system compartmentalizes and functionalizes the cytoplasm to ensure independent metabolic reactions. Recent studies have demonstrated that proteins in endomembrane system also have important roles in regulating fruit ripening and stress responses.

Certain endomembrane proteins, notably those resided on ER membrane, mitochondrial membrane and tonoplast are well received for their pleiotropic functions in signaling pathway or substance transport (Chen et al. 2020a; Eom et al. 2015; Jung et al. 2015). Among these endomembrane proteins, ethylene receptors (ETRs), CTR-like proteins (CTRs) and ETHYLENE INSENSITIVE 2 (EIN2) are involved in ET signaling, tonoplast sugar transporters (TST) regulate sugar transport, while vacuolar pyrophosphatase (V-PPase) and H^+^-ATPase (V-ATPase) are responsible for proton gradient formation, ultimately affecting fruit ripening and resistance to pathogens.

### ETR, CTR and EIN2

**Fig. 3:**
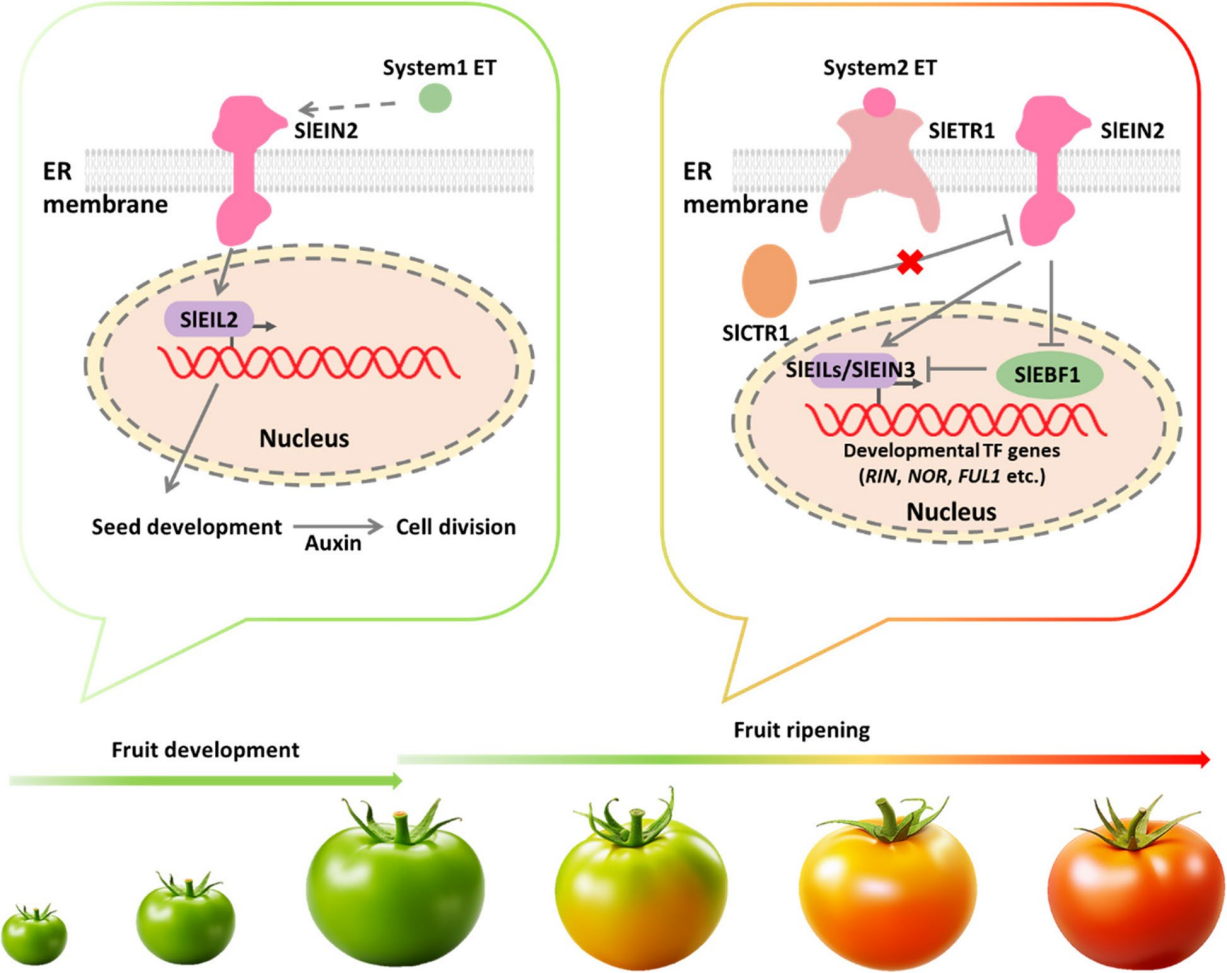
Proteins in endoplasmic reticulum (ER) membrane contribute to ethylene (ET) signaling and fruit development and ripening. Two systems of ET signaling, System-1 and system-2, control different aspects of fruit development and ripening. In system-1 ET signaling, ET controls the expression of SlEIN3/Ethylene Insensitive 3-Like transcription factors (EILs), particularly SlEIL2, by modulating the function of SlEIN2. SlEIL2 mediating cell division and fruit growth. In System-2 ET signaling, ET controls fruit ripening through a regulatory module comprising several key components: ethylene receptors (ETR), Constitutive Triple Responses (CTR), SlEIN2, SlEILs, and developmental transcription factors including Ripening-inhibitor (RIN), Non-Ripening (NOR), Fruitfull1 (FUL1), and EIN3 Binding F-box protein 1 (EBF1). Activated EILs transcriptionally trigger the expression of developmental transcription factor genes, thereby regulating fruit ripening by modulating the function of ripening-related genes

Hormones, particularly ET, are identified as key players in regulating fruit development and ripening (Brumos 2021; Xin et al. 2019). ET signaling pathways, facilitated by key membrane proteins located on the endoplasmic reticulum (ER) membrane, orchestrate various aspects of fruit development and ripening (Chen et al. 2020a). This cascade involves several critical components, including ETRs, CTRs and EIN2 (Fig. 3).

During fruit development and ripening, two systems of ET signaling, system-1 and system-2, exhibit distinct functions (Brumos 2021; Xin et al. 2019). System-1 ET signaling controls the expression of SlEIN3/Ethylene Insensitive 3-Like transcription factors (EILs), particularly *SlEIL2*, by modulating the function of SlEIN2. Association between *SlEIL2* and seed development underscores its significance in regulating fruit development (Huang et al. 2022). As seeds are rich sources of auxin, auxin may diffuse to surrounding tissues, mediating cell division and fruit development. In system-2 ET signaling, a regulatory module comprising ETRs, CTRs, SlEIN2, SlEILs, and ripening-related transcription factors [Ripening-inhibitor (RIN), Non-Ripening (NOR), Fruitfull1 (FUL1), and EIN3 Binding F-box protein 1 (EBF1)] controls fruit ripening (Huang et al. 2022). Notably, EIN2, EBF1, and EILs play pivotal roles in regulating fruit ripening. Suppression of *SlEIN2* expression resulted in delayed fruit ripening (Hu et al. 2010; Zhang et al. 2016), while loss-of-function mutation in *SlEIN2* led to delayed or interrupted fruit ripening, highlighting its central role in transmitting the ET signal (Huang et al. 2022; Ji and Guo 2013). Notably, the cessation of fruit maturation in *slein2* mutant was partially recovered by *slebf1*, rather than *slebf2* or *slebf3*, indicating that SlEBF1 was important in fruit ripening control (Huang et al. 2022). Further results showed that system-2 ET can directly trigger the expression of a series of ripening-related genes including *SlRIN*, *SlNOR* and *SlFUL1* via SlEILs. These findings highlight the contribution of EIN2-EBF1-EILs module to fruit ripening.

ETRs and CTRs regulate fruit ripening by modulating the function of EIN2. Tomato possesses seven ETRs, with differential contributions to fruit ripening regulation (Liu et al. 2015). Recent studies have verified the differential roles of the ETRs to the regulation of fruit ripening, although some ETRs may have redundant roles. For instance, mutants of *SlETR1*, *SlETR3* (also named as Never Ripe, NR), and *SlETR4* showed reduced ET sensitivity and delayed ripening (Mubarok et al. 2019; Okabe et al. 2011; Wilkinson et al. 1995). Conversely, the *SlETR4* RNAi fruit exhibited an early-ripening phenotype (Kevany et al. 2007a). However, the role of *SlETR5* and *SlETR6* mutants resulted in early ripening phenotypes (Kevany et al. 2007a, b; Mubarok et al. 2019), while overexpression or knockout of *SlETR7* had no detectable influence on fruit ripening (Chen et al. 2020b), possibly due to functional compensation by other ETRs. Similarly, CTRs, particularly SlCTR1, acted as negative regulators of ethylene signaling, as their silencing accelerated fruit ripening (Kamiyoshihara et al. 2022; Mata et al. 2018). CTRs physically interacted with ETRs and functioned as negative regulators of ethylene signaling. Tomato possesses four tomato *CTR1-like* genes (*SlCTR1*–*SlCTR4*) with differential expression patterns (Kamiyoshihara et al. 2022). *SlCTR1* was predicted to be the main regulator of ET signaling in fruit, and silencing *SlCTR1* accelerated fruit ripening (Fu et al. 2005). Recently, Kamiyoshihara et al. reported that *SlCTR1* and *SlCTR3* could interact with *SlETR4* to further regulate fruit ripening (Kamiyoshihara et al. 2022).

In the absence of ethylene, the ethylene receptor SlETR1 in tomato fruit interacts with SlCTR1 (Kamiyoshihara et al. 2022). This interaction leads to the suppression of the ethylene response by keeping SlEIN2 in an inactive state. Consequently, the downstream ethylene-responsive transcription factors, SlEIN3 and Ethylene Insensitive 3-Like (EILs), remain inactive, preventing the expression of ripening-related genes (Huang et al. 2022). However, in the presence of ethylene, SlEIN2 is activated. The activated SlEIN2 controls the expression of *SlEIN3* and *SlEILs*, which in turn triggers the expression of a series of ripening-related genes, including *SlRIN*, *SlNOR*, and *SlFUL1* (Huang et al. 2022). These genes play crucial roles in the regulation of tomato fruit ripening. The subcellular localization of membrane proteins can affect their interactions with other proteins and molecules. In *Arabidopsis*, more detailed ethylene signaling pathway have been presented. In the absence of ethylene, ETR interacts with CTR1, which phosphorylates and thereby inactivates EIN2 (Ju et al. 2012). This phosphorylation prevents the downstream ethylene response by keeping EIN2 inactive. When ethylene is present, both ETR and CTR1 are inactivated, leading to the dephosphorylation and cleavage of the C-terminus of EIN2 (Qiao et al. 2012). The released C-terminal fragment of EIN2 then translocate from the endoplasmic reticulum (ER) to the nucleus (Wen et al. 2012). In the nucleus, this fragment activates the EIN3 and EIN3-LIKE1 (EIL1) transcription factors (Qiao et al. 2012; Wen et al. 2012). These transcription factors regulate the expression of downstream ethylene-responsive genes, modulating various growth and stress response processes in plants. By understanding these pathways, researchers can better comprehend how ethylene regulates ripening and other physiological responses in plants, providing potential targets for genetic and chemical modulation to improve fruit yield and quality.

Aside from its functions in regulating fruit ripening, ET signaling also plays a role in fruit defense against pathogens. Disruption of ET signaling in kiwifruit reduced fungal decay, while transgenic plants with altered ET signaling exhibited varied susceptibility to pathogen infections (Lin et al. 2008; Xia et al. 2020). For instance, the disturbance of ET signaling in kiwifruit using 1-MCP reduced the incidence of *Phomopsis* sp. decay (Xia et al. 2020), while the fruit from *LeCTR2* transgenic plants exhibited more severe *B. cinerea* infection than wild type (Lin et al. 2008). Although the roles of ETRs, CTRs, and EIN2 in ET signaling during fruit ripening is well understood, their involvement in pathogen responses still requires further investigation. Insights from studies in other crops imply potential involvement of EIN2 in fruit defense against pathogens (Low et al. 2022; Qiu et al. 2022). The *Arabidopsis ein2* mutant showed a higher resistance to *Fusarium* head blight (FHB) than the wild type plants, whereas the complementation with a barley *HvEIN2* gene restored the FHB susceptibility phenotype of *Arabidopsis ein2* mutant, implying functional equivalence of the barley HvEIN2 ortholog (Low et al. 2022). Moreover, the knock-out of *OsEIN2* impaired rice resistance to *Magnaporthe oryzae* by interfering with the activation of ET signaling (Qiu et al. 2022).

### Tonoplast sugar transporters

Soluble sugars are ubiquitous in fleshy fruit, with their accumulation being a major quality trait of fruit (Shammai et al. 2018). Meanwhile, sugars also serve as signaling molecules regulating various physiological processes in plants, including responses to biotic and abiotic stresses. Additionally, sugar signaling influences fruit development by regulating processes, such as cell division, expansion, and differentiation, as well as the accumulation of secondary metabolites and flavor compounds (Jia et al. 2013). The contents of soluble sugars in diverse subcellular localizations depends on specific transporters on tonoplast or PM (Eom et al. 2015; Jung et al. 2015). This part mainly focuses on tonoplast sugar transporters (TSTs) involved in regulating fruit ripening, quality formation, and cold stress responses.

In fleshy fruit, several *TST* genes have been demonstrated to modulate sugar accumulation and fruit ripening. For example, *ClTST2* was identified as a quantitative trait locus (QTL) for sugar content in watermelon (Ren et al. 2018). Overexpression of *ClTST2* in watermelon fruit resulted in higher sugar accumulation than control, as ClTST2 transport both sucrose and hexoses. Similarly, overexpression of *CmTST2* increased sugar accumulation in melon fruit (Cheng et al. 2018). Notably, ectopic expression of *ClTST2* or *CmTST2* in strawberries affected fruit quality by increasing sugar accumulation and delaying the transition of fruit color. In apple fruit, sugar accumulation in vacuoles was mediated by the coordinated action of two classes of tonoplast sugar transporters (Li et al. 2022; Zhu et al. 2020). MdERDL6-1, a tonoplast H^+^/glucose symporter, mediated glucose efflux to the cytosol (Zhu et al. 2020). However, the concentrations of glucose, fructose and sucrose increased in transgenic apple and tomato fruit overexpressing *MdERDL6-1*. To explore the underlying mechanisms, Zhu et al. (2020) performed RNA-sequencing in transgenic apple lines overexpressing *MdERDL6-1*. Further analysis revealed that the up-regulation of tonoplast sugar transporters *TST1* and *TST2* in apple and tomato was associated with the overexpression of *MdERDL6-1*. Knockout of *SlTST1* and *SlTST2* in the *MdERDL6-1*–overexpressed tomato abolished the positive effect of *MdERDL6-1* on sugar accumulation, indicating that the function of MdERDL6-1 in sugar accumulation may depend on its regulation on the expression of *TST1* and *TST2*. Indeed, another study reported the crucial roles of MdTST1 and MdTST2 in sugar accumulation in tomato fruit (Li et al. 2022). In a recent study, a sophisticated regulatory mechanism, the SnRK2.3-AREB1-TST1/2 cascade, was reported (Zhu et al. 2023). Specifically, MdERDL6-1 positively regulated the expression of *SnRK2.3*, which subsequently phosphorylated the MdAREB1 transcription factor and activated the expression of *MdTST1* or *MdTST2*, thereby promoting sugar accumulation. Additionally, two low temperature-induced C-repeat binding factors, MdCBF1 and MdCBF2, activated the expression of *MdTST1* or *MdTST2*, suggesting that the *MdCBF1/2*-*MdTST1/2* module affected apple defense against cold stress (Li et al. 2024a). Similarly, CsTST1 responded to various biotic stresses, such as drought and cold (Huang et al. 2020). As currently available studies on TSTs in fruit mainly focus on their functions related to fruit quality, further efforts are required to identify and characterize TSTs that function in fruit defense against stresses.

### Other endomembrane proteins

Some other endomembrane proteins are also involved in fruit ripening and defense responses to pathogen invasions.

### Anion channel proteins

Mitochondria, known as the center of energy metabolism in cells, have been implicated in defense responses against pathogen invasions (Tian et al. 2013). An anion channel protein localized in the outer membrane of mitochondria, MdVDAC2, was found to affect the defense response of apple cells against *Botryosphaeria dothidea* (Xin et al. 2023). Overexpression of *MdVDAC2* in apple fruit cells led to more severe cell death and inhibited the infection by *B. dothidea*, indicating its contribution to pathogen resistance in apple fruit by inducing cell death.

Potassium is essential for plant growth and exhibits critical effects on fruit quality (Conde et al. 2006; Nieves-Cordones et al. 2016). FaTPK1, a tonoplast-localized two-pore potassium channel in strawberry, regulates fruit ripening and quality formation (Wang et al. 2017). Modulating the expression of *FaTPK1* affected fruit ripening, with up-regulation accelerating ripening and down-regulation suppressing ripening. In addition, following *B. cinerea* inoculation, *FaTPK1*-RNAi fruit displayed a resistant phenotype compared to the wild type fruit. Further results indicated that the regulation of *FaTRK1* on fruit ripening and resistance may depend on its activation of the expression of *GAL6* (beta-galactosidase), *PG1* (polygalacturonase), *XYL2* (D-xylulose reductase), *SUT1* (sucrose transporter), *CHI* (chalcone flavanone isomerase) and *CHS* (chalcone synthase).

### Vacuolar transporters and vacuolar proton pumps

The transportation of secondary metabolites during fruit ripening, especially organic acids, is an important process contributing to the changes in fruit pH, thereby forming distinct flavors of various fruit (Etienne et al. 2013; Shi et al. 2018). Malate is the predominant organic acid in many fruit (Colaric et al. 2005; Sweetman et al. 2009). Vacuolar transporters and vacuolar proton pumps are responsible for malate transport. By means of genome-wide association studies (GWAS), *SlALMT9* was found to be involved in malate accumulation in tomato fruit (Ye et al. 2017). Further results showed that a 3-bp indel in the *SlALMT9* promoter promoted fruit malate accumulation by disturbing the binding of SlWRKY45 to the promoter of *SlALMT9*. Identically, several vacuolar malate transporter genes have also been identified in apple, among which *MdMa1* and *MdALMTII* are associated with low fruit acidity, while *Ma10* contributes to malate accumulation (Bai et al. 2012; Jia et al. 2018; Li et al. 2020a; Ma et al. 2018). The function of MdALMT9 depends on its C-terminal domain (Li et al. 2020a). Vacuolar pyrophosphatase (V-PPase) and H^+^-ATPase (V-ATPase), two primary vacuolar proton pumps responsible for generating the proton electrochemical gradient, are required for the activities of malate transporters (Hu et al. 2016). However, the regulatory mechanism of the activity of these vacuolar membrane proteins is rarely reported, it was reported that MdMYB1 and MdMYB73 directly modulated the vacuolar proton pumps in apple by activating the expression of *MdVHA* and *MdVHB*, thereby improving the activities of malate transporters (Hu et al. 2016, 2017). Moreover, MdBT2, a BTB-BACK-TAZ domain protein, negatively regulated the malate accumulation in apple fruit by facilitating the ubiquitination of MdMYB73 (Zhang et al. 2020a). However, whether MdBT2 influences malate accumulation by MdMYB1 is still unclear.

## Future perspectives

**Fig. 4:**
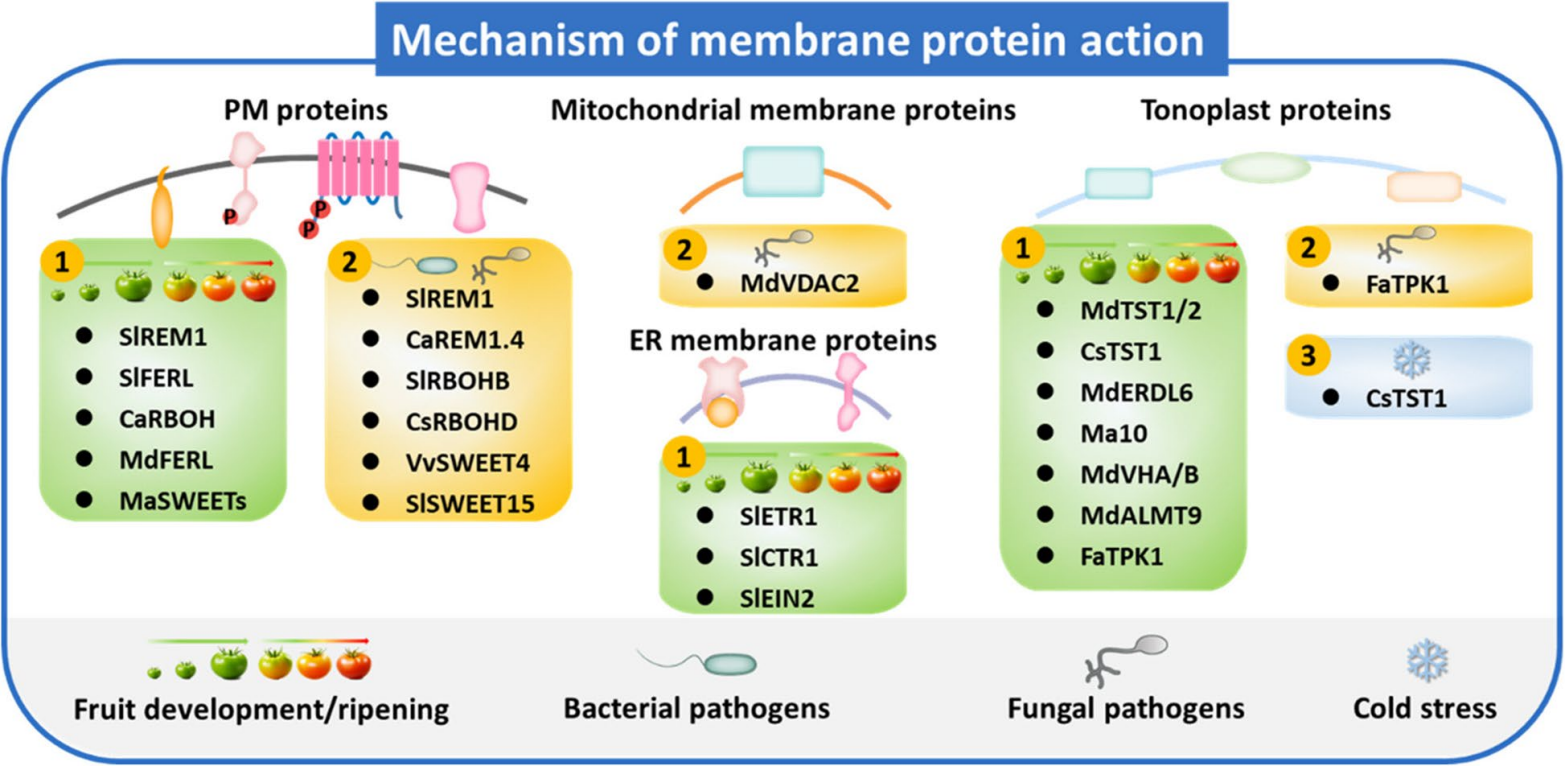
Functions of membrane proteins in regulating fruit development/ripening (1), fruit defense against pathogens (2), and cold stress (3). PM, plasma membrane; ER, endoplasmic reticulum

The understanding of membrane protein functions in perceiving environmental signals and regulating fruit ripening and stress responses has made rapid progress, thus providing potential targets for postharvest control of fruit quality and resistance against stresses (Fig. 4). However, there are still some open questions to be addressed:

First, it is critical for the survival of plants to maintain the delicate balance between the activation and inactivation of plasma membrane protein-mediated immunity upon pathogen attacks (Couto and Zipfel 2016). Although previous studies have highlighted the roles of pattern recognition receptor (PRR) activation and attenuation in modulating immune responses, the mechanisms in balancing fruit ripening and immunity are still far from being understood (Duplan and Rivas 2014; Frescatada-Rosa et al. 2015). Therefore, more efforts should be made to understand the underlying mechanisms regulating this balance, such as ubiquitination-mediated degradation, endocytosis and involvement of non-coding RNAs (ncRNAs), for improving plant resistance to pathogens (Chen et al. 2024; Cui et al. 2018; Liu et al. 2024; Zou et al. 2018), which may contribute to developing strategies to enhance fruit resistance to pathogens while avoiding overactivation of immune responses.

Second, the change in subcellular location of membrane proteins inevitably affects their functions (Garg and Kühn 2022). Environmental factors, such as light, temperature and pathogens, may induce the endocytosis of membrane protein (as previously reported for regulator of G protein signaling 1, SlRGS1) from plasma membrane, thereby altering their functions and affecting resistance to *Pseudomonas syringae* (Wang et al. 2022a). It may undoubtedly accelerate practical utilization of membrane proteins in regulating fruit ripening or resistance to elucidate the correlation between subcellular localization of membrane proteins and their functions and further explore effective external factors that modulate such correlations.

Additionally, as an ultimate goal of scientific research is to transform research achievements into practical application, attempts have been made in utilizing plasma membrane proteins to regulate fruit ripening or enhance resistance, such as the mutants with reduced ET sensitivity exhibited extended shelf life, the synthetic peptides delaying tomato ripening (Kessenbrock et al. 2017; Mubarok et al. 2015), as well as transgenic plants expressing membrane proteins showed enhanced resistance to pathogens (Mitre et al. 2021; Piazza et al. 2021; Tripathi et al. 2014). However, there is an urgent need to evaluate the safety of these applications before putting into practice.

## Data Availability

Not applicable.

## Abbreviations

ABA: Abscisic acid
CTRs: CTR-like proteins
EBF1: EIN3 Binding F-box protein 1
EILs: EIN3/Ethylene Insensitive 3-Like transcription factors
EIN2: Ethylene Insensitive 2
ER: Endoplasmic reticulum
ET: Ethylene
ETRs: Ethylene receptors
FERL: FERONIA Like
FUL1: Fruitfull1
MAPK: Mitogen-activated protein kinase
MSTs: Monosaccharide transporters
ncRNAs: Non-coding RNAs
NLS: Nuclear localization signal
NLS: Nuclear localization signal
PG1: Endopolygalacturonase1
PM: Plasma membranes
PRR: Pattern recognition receptor
QTL: Quantitative trait locus
RBOHB: Respiratory Burst Oxidase Homolog
REMs: Remorins
RIN: Ripening-inhibitor
RLKs: Receptors-like kinases
SAMS: *S*-Adenosyl-Met synthetase
SOTs: Sorbitol transporters
SUTs: Sucrose transporters
TSTs: Tonoplast sugar: transporters
V-PPase: Vacuolar pyrophosphatase
V-ATPase: H^+^-ATPase
